# Effects of zinc incorporation on hierarchical ZSM-11 catalyst for methanol conversion

**DOI:** 10.1007/s13203-015-0120-3

**Published:** 2015-07-25

**Authors:** Xiaojing Meng, Chen Chen, Jianwei Liu, Qiang Zhang, Chunyi Li, Qiukai Cui

**Affiliations:** 1 State Key Laboratory of Heavy Oil Processing, China University of Petroleum (East China), No. 66 West Road, Qingdao, China; 2 Dagang Petrochemical Company, Tianjin, China

**Keywords:** Hierarchical ZSM-11, Methanol, Zinc, Acid site, Structure damage

## Abstract

**Abstract:**

Hierarchical ZSM-11 and Zn-ZSM-11 catalysts were used in this study. The effects of two methods (direct synthesis and impregnation) of zinc incorporation on methanol conversion were investigated in a continuous-flow isotherm fixed-bed reactor. XRD, SEM, BET, FTIR, and XRF analytical results revealed that the introduction of zinc through direct synthesis generated new Brønsted acid sites that could tune the ratio of light olefins. The damage to the framework structure after zinc incorporation restrained the aromatization, dehydrogenation, and decomposition of methanol. The extent of this impact determined the degree of deactivation behaviors. Thus, the yield of propene and butene was enhanced through the direct synthesis method (2 % ZnZ11-C, 4 % ZnZ11-C), and the sample 4 % ZnZ11-C displayed a fast deactivation.

**Graphical Abstract:**

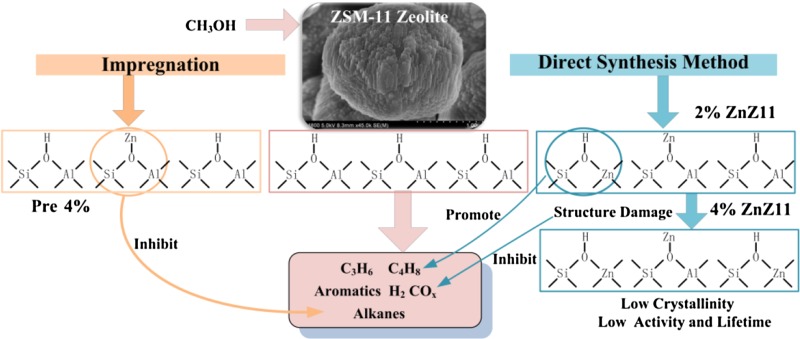

## Introduction

Methanol-to-hydrocarbon (MTH) technology, mainly the methanol-to-gasoline (MTG) and methanol-to-olefin (MTO) reactions, are regarded as a competitive route to convert coal or natural gas into high-octane gasoline and chemicals because of the shortage of petroleum resources. Studies indicate that the distribution of products obtained by zeolitic methanol conversion strongly depends on the acidity (acid strength and number of acid sites) and channel structure of zeolite [1].

Recent studies modulating the product distribution in methanol conversion have mainly focused on ZSM-5 zeolite with the incorporation of several metal species, such as Ag/ZSM-5 [2], Cu/Zn/HZSM-5 [3], and Ga_2_O_3_/HZSM-5 [4]. The species above mainly exhibit good methanol-to-aromatics (MTA) ability and high benzene–toluene–xylene (BTX) yield. Mohammad Rostamizadeh et al. found that the Mn and P promoters can control the side reactions and reduce the by-products to improve the selectivity of propene [5]. Moreover, zinc species are known to play an essential role in the enhancement of the aromatics selectivity. Ono et al. [6] concluded that zinc ions were important for the dehydrogenation of alkenes to aromatics. Simultaneously, the presence of zinc ions promoted the decomposition of methanol. Ni et al. [7] prepared nano-sized H[Zn, Al]ZSM-5 zeolite through the direct synthesis procedure. The direct synthesis method was found to be beneficial for the dispersion of Zn species. In addition, the nanostructure could control methanol decomposition and avoid deep aromatization. The above-mentioned studies indicate that the role of Zn species in zeolite is determined by different channel structures and acidity of zeolite.

Recently, our research group has synthesized a hierarchical ZSM-11 zeolite through a simple and low-cost method [8]; the material features intercrystalline mesoporous and rod-like crystal intergrowth morphology. This zeolite has been successfully produced on an industrial scale and its derivatives displayed excellent activity in methanol and glycerol conversion reactions [9–12]. The nanostructure, mesopores, and low sinuosity make ZSM-11 favorable for facile diffusion of primary products and coke precursors; thus, it can reduce secondary reactions and prolong the catalyst lifetime [13]. In a previous study, it was found that ZSM-11 catalyst indeed promoted the production of propylene and gasoline in methanol conversion [14]. The role of zinc in the structure, properties, and reaction performance of this zeolite is still unclear; thus, the modification of zinc species on ZSM-11 zeolite through two methods, namely, direct synthesis and impregnation, is investigated.

## Experimental

### Catalyst preparation

Hierarchical ZSM-11 zeolite was prepared according to the method described in Refs. [10, 11]. In short, the synthesis was as follows: 2.54 g NaOH and 2.26 g Al_2_(SO_4_)_3_·18H_2_O were dissolved in 20 g H_2_O. Then a mixture composed of 33.05 g silica sol, 1.16 g tetrabutylammonium bromide (TBABr), and 20 g H_2_O was added, followed by addition of ZnO powder. The molar composition of the hierarchical ZSM-11 zeolite mixture was Na_2_O:Al_2_O_3_:ZnO:SiO_2_:(TBA)_2_O:H_2_O = 9.0:1.0:*X* (*X* = 0.0, 5.0, 9.0):65:0.5:1300. The gel was transferred into a teflon-lined stainless-steel autoclave. The crystallization was first carried out at 90 °C for 24 h and then heated to 170 °C for 8 h. The product was filtered, washed, dried, and then calcined at 550 °C in air for 3 h with a heating rate of 10 °C/min. Then the zeolites were turned into the H form by three consecutive ion exchanges in 1 mol/L NH_4_NO_3_ solution at 80 °C for 2 h. The resultant zeolite was designated as Parent, 2 % ZnZ11, and 4 % ZnZ11 based on the XRF analysis result. Pre 4 % zeolite was obtained through impregnation of an aqueous solution of Zn(NO_3_)_2_·6H_2_O at a Zn/zeolite ratio of 0.04.

Catalysts were prepared with 50 wt% of kaolin, 35 wt% of zeolite and 15 wt% of colloidal silica as a binder (40 wt% SiO_2_). The solid solution was dried and calcined at 700 °C for 2 h. The catalysts were denoted as Parent-C, 2 % ZnZ11-C, 4 % ZnZ11-C, and Pre 4 %-C, respectively.

### Catalyst characterization

Bulk crystalline phases of the catalysts were determined by X-ray diffraction (XRD) on Philips X’Pert PRO MPD diffractometer (PANalytical Company, The Netherlands). The morphology and structure of the solids were investigated with scanning electron microscopy S-4800 (Hitachi Company, Japan). Textural parameters of the samples were determined by nitrogen adsorption isotherms using Quantachrome Autosorb iQ apparatus. X-ray fluorescence spectroscopy (Axios) was used to analyze the composition of the samples. The characteristic vibration bands of the zeolites were measured by FTIR on a Nexus Model Infrared Spectrophotometer (Nicolet Co, USA). The samples were pretreated in N_2_ flow for 1 h at 500 °C in vacuum. Pyridine Fourier-transform infrared spectroscopy (Py-FTIR) measurements were performed to study the acid properties by NEXUS FTIR. The coking amount was calculated from a TG–DTA curve measured on a NET-ZSCH Proteus STA449C in air.

### Catalytic testing

**Fig. 1:**
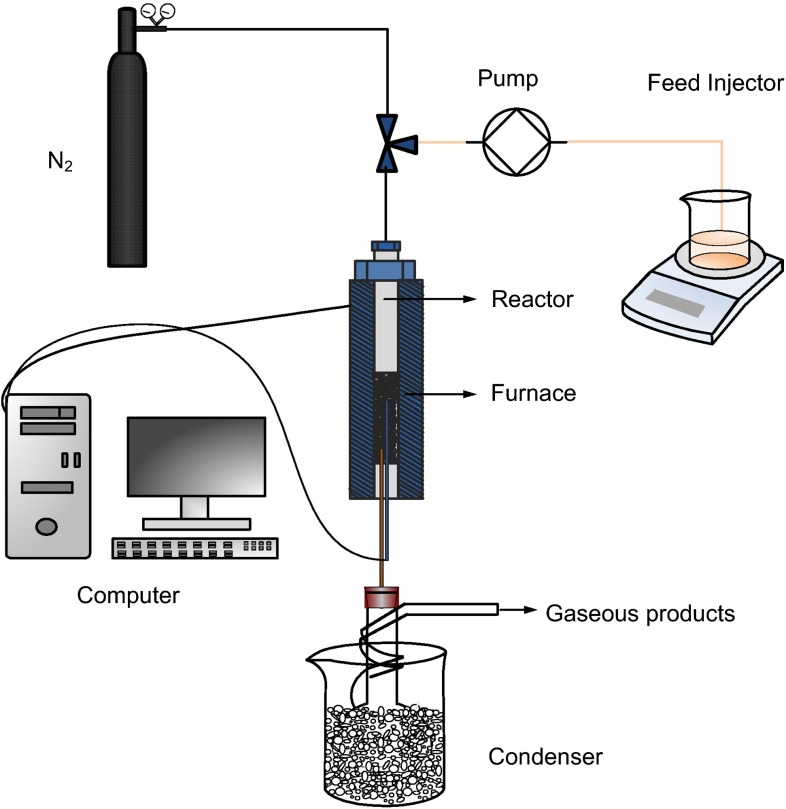
Schematic diagram of the catalytic test unit for methanol conversion

The catalytic testing was carried out at 450 °C in a fixed-bed microreactor under atmospheric pressure. The setup for catalyst test is shown in Fig. 1. The catalyst loading was 2.0 g (40–60 mesh) and the weight hourly space velocity (WHSV) for pure methanol was 5.53 h^−1^.

The composition of gaseous products was analyzed using a Bruker 450-GC gas chromatography (GC) with a TCD detector to analyze the content of hydrogen, nitrogen, and carbon oxide, and an FID detector column to determine the composition of hydrocarbons. Liquid products were analyzed on Agilent 6820 gas chromatograph (GC) equipped with HP-INNOWAX capillary column (30 m × 0.32 mm × 0.25 μm) and a flame ionization detector (FID) using ethanol as an internal standard. Both methanol and DME were regarded as reactants for calculation.

## Results and discussion

**Fig. 2:**
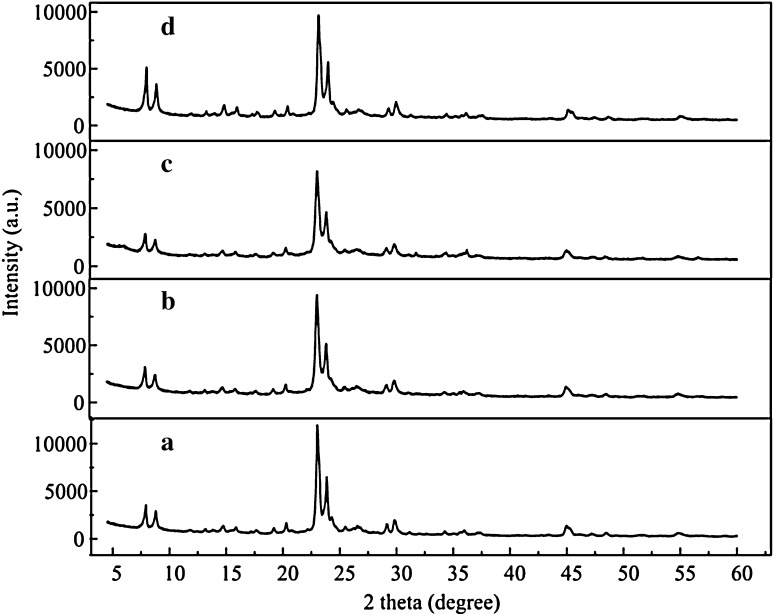
XRD patterns of the zeolites: *a* Parent; *b* 2 % ZnZ11; *c* 4 % ZnZ11; *d* Pre 4 %

**Fig. 3:**
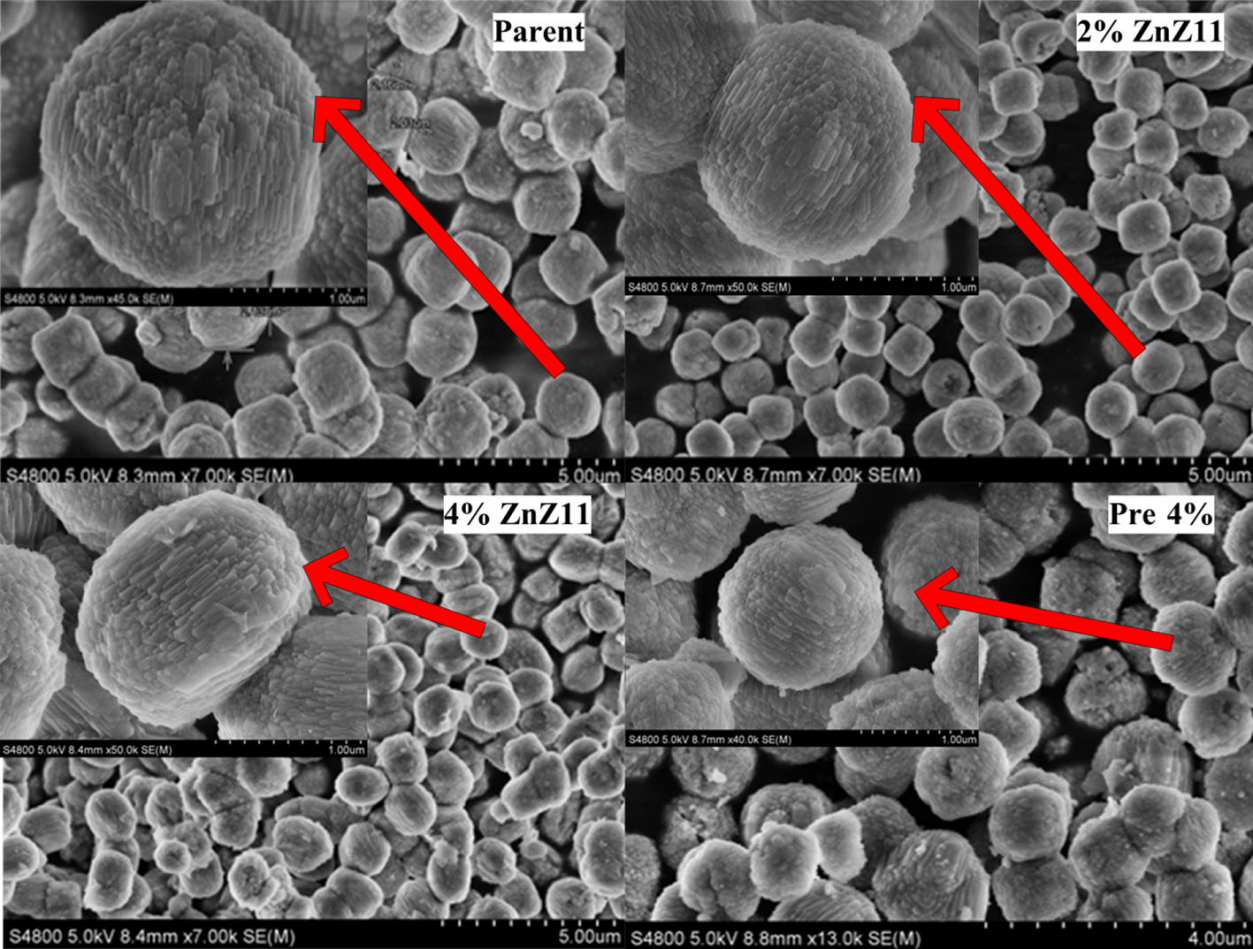
SEM images of the zeolites

Figure 2 exhibits typical diffraction peaks indexed to [501] and [303] crystal planes, corresponding to the MEL framework structure [15]. No diffraction peaks of zinc oxide crystallites were observed, indicating that the pure phase of the samples were obtained and zinc species were highly dispersed on the surface or in the skeleton of the zeolite. Si/Al ratio of all zeolites measured by XRF was approximately 28 ± 1, and zinc content in Pre 4 % was close to 4 wt%. Figure 3 presents the structure of ZSM-11 zeolite, including nanorods, which grafted and aggregated together to form spheroidal particles of approximately 1–2 μm in diameter. The smooth and original spheroidal forms of the crystal surface tended to be oval through direct synthesis, implying that zinc was incorporated in the formation of zeolite and thus affected the morphology [11].

**Fig. 4:**
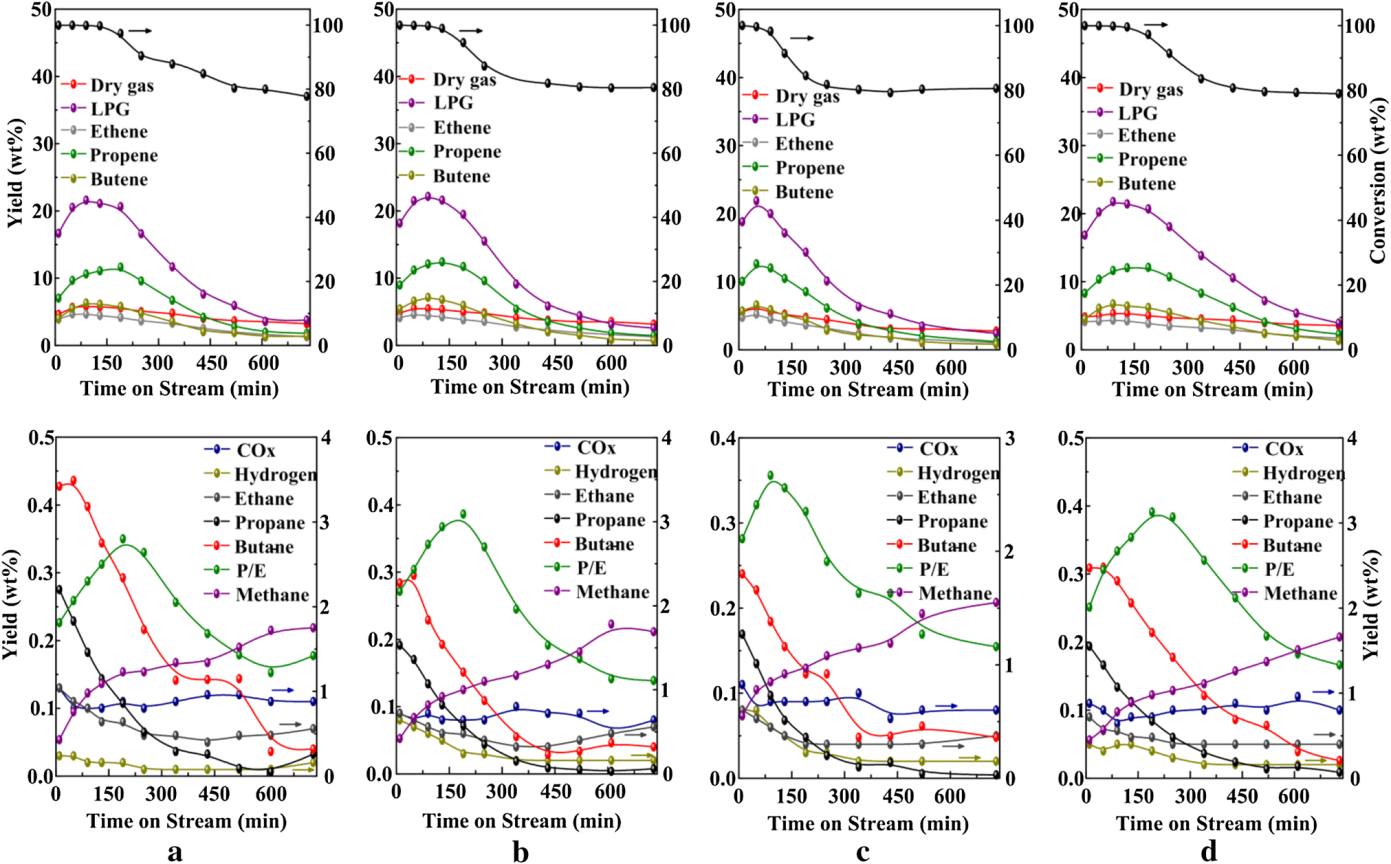
Methanol conversion and product distribution vs. time over various catalysts: **a** Parent-C; **b** 2 % ZnZ11-C; **c** 4 % ZnZ11-C; **d** Pre 4 %-C

**Fig. 5:**
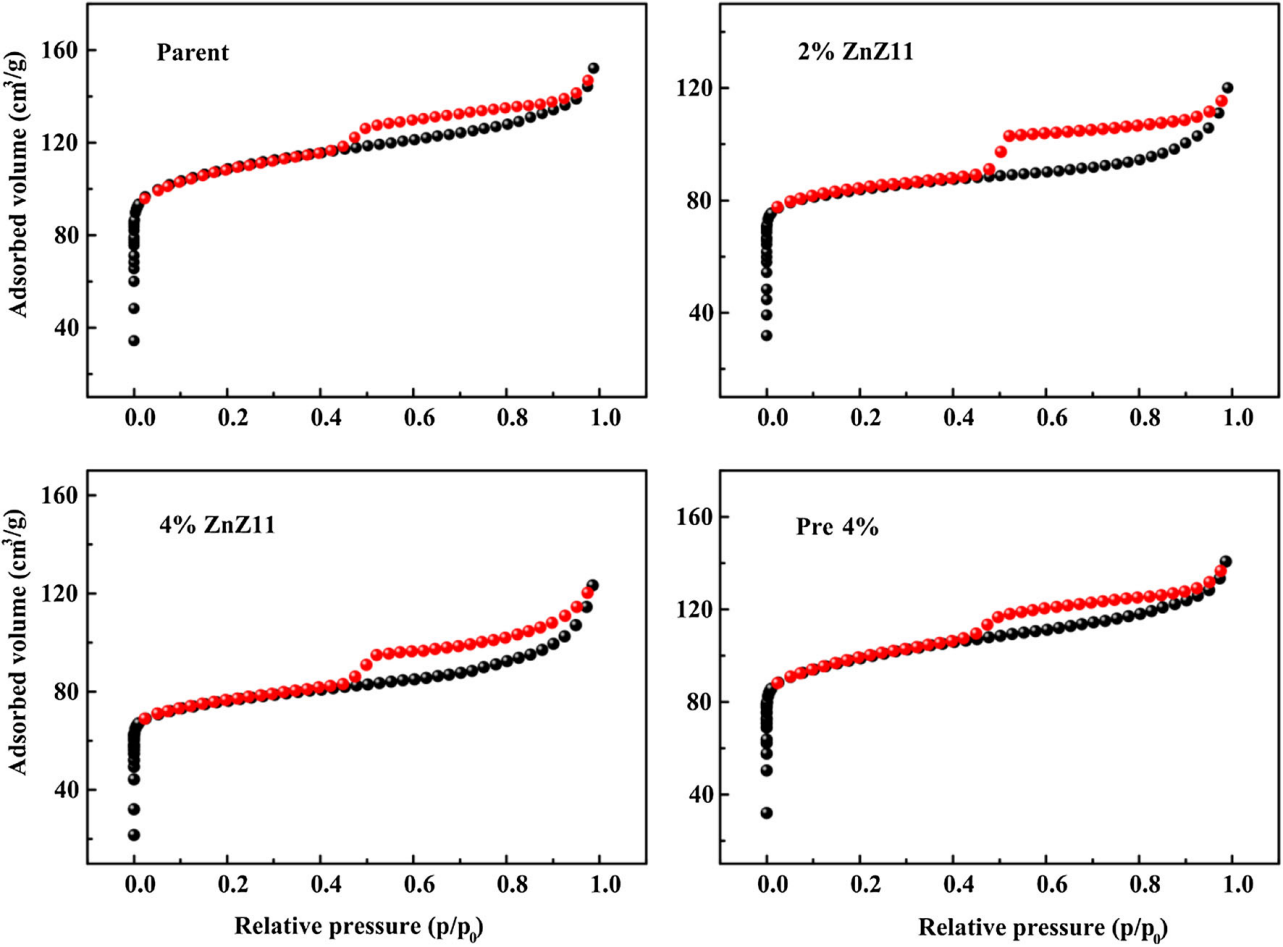
The N_2_ adsorption–desorption isotherms of the zeolites

**Fig. 6:**
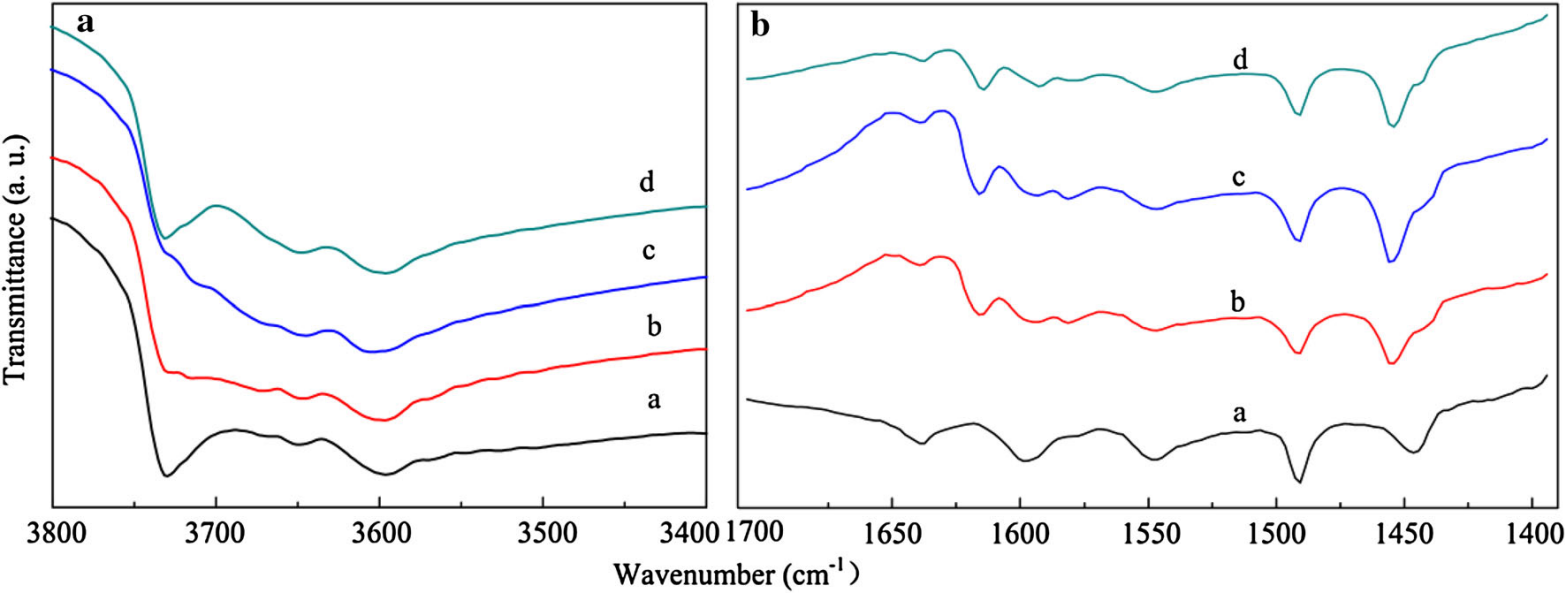
FTIR spectra in the OH-stretch region after activation at 500 °C (**A**) and pyridine adsorption (**B**) of the zeolites: *a* Parent; *b* 2 % ZnZ11; *c* 4 % ZnZ11; *d* Pre 4 %

The catalysts were tested in MTO reaction under the same reaction conditions. The results in Fig. 4 show that all catalysts demonstrate almost 100 % of initial methanol conversion. However, the deactivation rates were quite different. Parent-C, Pre 4 %-C, and 2 % ZnZ11-C retained a methanol conversion of approximately 90 % after 2 h on stream. By contrast, reduction in methanol conversion for 4 % ZnZ11-C was accelerated at 1.5 h. N_2_ adsorption–desorption isotherms (Fig. 5) and physical properties (Table 1) of the samples revealed that the difference for Pre 4 %-C and 2 % ZnZ11-C was mainly located in pore volume (0.22 and 0.19 cm^3^/g, respectively) and surface area (375 and 329 m^2^/g, respectively). No significant influences were displayed for the catalytic lifetime. In addition, the amount of Brønsted acid sites for Pre 4 %-C shown in Fig. 6 decreased drastically compared with Parent-C. The change of acid sites in the present range also hardly affected the deactivation behaviors. Thus, the relative low pore volume, surface area, and acid amount in the study were not crucial for the rapid deactivation of 4 % ZnZ11-C. The negative performance might be ascribed to the severe damage to the framework, as reflected by the poor crystallinity.

**Table 1:** Textural properties and relative crystallinity of the zeolites

Sample	Surface area (m^2^/g)			Pore volume (cm^3^/g)			Relative crystallinity (%)
	*S*_Ext_	*S*_Micro_	*S*_BET_	*V*_micro_	*V*_meso_	*V*_Total_	
Parent	72	341	413	0.138	0.098	0.236	100
2 % ZnZ11	37	293	329	0.114	0.072	0.186	88
4 % ZnZ11	50	243	293	0.097	0.094	0.191	70
Pre 4 %	71	304	375	0.123	0.095	0.218	94

Product distribution on these catalysts was observed to be extremely similar. As seen, ZSM-11 catalyst was notably excellent for converting methanol into propene and petroleum-range boiling products. However, the yield of BTX at 2 h on stream significantly decreased after direct incorporation of zinc (Table 2). Machado et al. found that iron species in [Fe, Al]-ZSM-5 could be extracted from the structure by severe calcination, which resulted in low yield of liquid hydrocarbons and short catalytic lifetime in the conversion of ethanol or methanol [16]. Our result agreed well with Machado’s findings. The poor aromatization performance and short catalytic lifetime of the catalysts by direct synthesis could be due to the partial removal of Zn species upon severe calcination and subsequent damage to the zeolite framework. However, Zn species were located on the outer surface of the sample through impregnation, and thus this had little effect on Pre 4 %. Consequently, the production of hydrogen was inhibited for weak dehydrogenation and the decomposition of methanol was suppressed simultaneously by direct synthesis, which was linked to the lower yield of carbon oxides [6]. In addition, it was interesting to note that 4 % ZnZ11-C was more favorable for the formation of propene and butene than Pre 4 %-C under the same zinc content.

**Table 2:** Product distribution for methanol conversion on various catalysts

Catalysts	Conversion (wt%)	Yield (wt%)								Y_BTX_^a^ (wt%)
		CH_4_	C_2_	C_3_	C_4_	C_6_H_6_	C_7_H_8_	C_8_H_10_	C_9_H_12_	
Parent-C	99.9	1.0	4.6	10.6	6.3	0.2	2.0	5.7	1.6	7.8
2 % ZnZ11-C	99.8	0.8	4.4	12.0	7.1	0.2	1.7	4.0	0.7	5.8
4 % ZnZ11-C	98.2	0.8	4.4	12.2	7.2	0.1	1.2	3.6	0.6	4.9
Pre 4 %-C	99.8	0.8	4.4	11.0	6.73	0.2	1.9	5.2	1.5	7.3

Reaction conditions: 450 °C, WHSV = 5.53 h^−1^. Data obtained at 2 h TOS

^a^The yield of BTX hydrocarbons

**Fig. 7:**
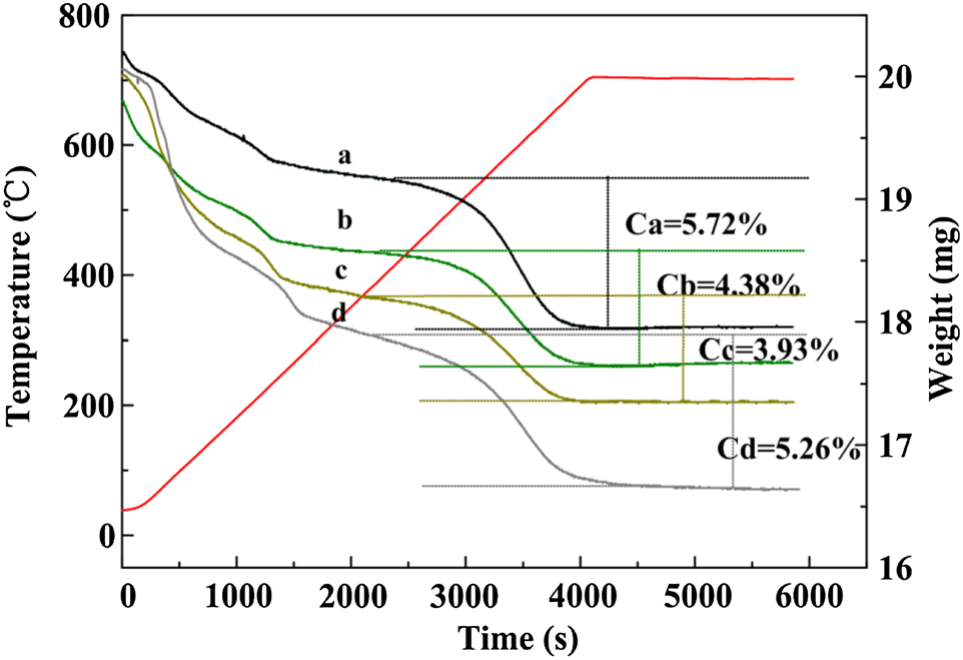
TGA Profiles of coked catalysts. The weight loss after 300 °C was representative of the amount of coke: *a* Parent-C; *b* 2 % Zn11-C; *c* 4 % Zn11-C; *d* Pre 4 %-C

**Fig. 8:**
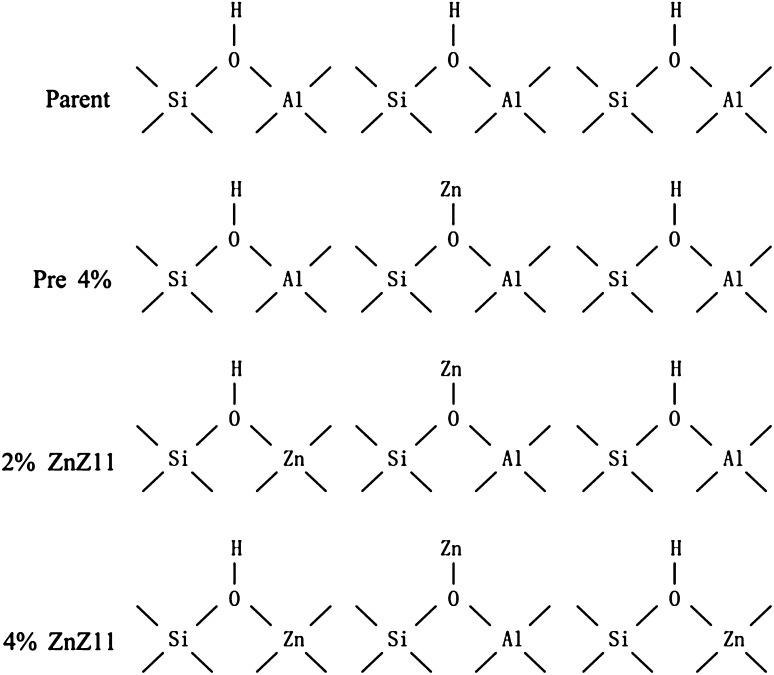
Change in the structure of modified acid sites during introduction of Zn

The determination of acid sites by adsorption of pyridine was employed to observe the correlation between the acid sites and product distribution. Moreover, the nature of acid sites was derived from the IR spectra of the OH groups in the zeolites. Pyridine-IR spectra (Fig. 6b) revealed that the increase of Lewis acid amount was a result of new strong Lewis acid sites generated, which could be observed in the new signal at 1616 cm^−1^ and double bands at 1450 cm^−1^ [17, 18]. Meanwhile, the intensity of the band at 3596 cm^−1^ associated with bridging hydroxyl groups (Si(OH)Al) was declined for zinc introduction due to the partial exchange of acidic hydroxyl groups by zinc ions [18]. This phenomenon could give an explanation for the high resistance to coking for zinc-introduced catalysts (Fig. 7) [19]. Nevertheless, compared with Pre 4 % and 2 % ZnZ11, 4 % ZnZ11 provided more Brønsted and Lewis acid sites. The extra-framework alumina and zinc formed because of the structure damage and the formation of O^−^–Zn^2+^–O^−^ species could be responsible for increasing Lewis acid sites [20]. New Brønsted acid sites might be generated for Zn species entering and participating in the formation of the zeolite framework. Moreover, the shift to higher wavenumber of around 3610 cm^−1^ for Zn directly introduced catalysts was accompanied by a high yield of propene and butene. In addition to the fact that the Zn^2+^ cation in bulk ZnO was in the [ZnO_4_]^6−^ tetrahedral unit [21], ZnO_4_ tetrahedral units would bond with surrounding silicate tetrahedral (Fig. 8) [22]. Therefore, the distribution of light olefins might be possibly related to the formation of Brønsted acid sites (Si(OH)Zn). Nonetheless, the newly formed Brønsted acid sites could not counteract the effect of damaged structure and exchange of zeolitic protons with the zinc ions, resulting in the decrease in the total amount of Brønsted acid sites and secondary reactions compared with Parent-C. The intensity of band at 3740 cm^−1^ (Fig. 6a) assigned to the isolated silanol groups (SiOH) located on the external surface of zeolite dropped sharply through direct synthesis due to low external surface illustrated in Table 1 and the formation of ZO–Zn–O–Si species at the expense of silanol groups by zinc incorporation [23].

## Conclusions

In this study, the hierarchical ZSM-11 and Zn-ZSM-11 catalysts were applied in MTO reactions. Two methods for zinc incorporation were compared. Experimental results demonstrated that the yield of alkanes and coke declined after the introduction of Zn because of decreasing Brønsted acid sites. The direct synthesis method generated new Brønsted acid sites from (Si(OH)Zn), which favored the formation of propene and butene. The structural damage to catalysts due to the direct incorporation of zinc species could be disadvantageous to the formation of aromatics, hydrogen, and carbon oxides. The deactivation behaviors might be attributed to the extent of structure damage.
